# Obesity Related Alterations in Plasma Cytokines and Metabolic Hormones in Chimpanzees

**DOI:** 10.1155/2014/856749

**Published:** 2014-09-18

**Authors:** Pramod Nehete, Elizabeth R. Magden, Bharti Nehete, Patrick W. Hanley, Christian R. Abee

**Affiliations:** Department of Veterinary Sciences, The University of Texas MD Anderson Cancer Center, Bastrop, TX 78602, USA

## Abstract

Obesity is characterized by chronic low-grade inflammation and serves as a major risk factor for hypertension, coronary artery disease, dyslipidemias, and type-2 diabetes. The purpose of this study was to examine changes in metabolic hormones, inflammatory cytokines, and immune function, in lean, overweight, and obese chimpanzees in a controlled environment. We observed increased plasma circulating levels of proinflammatory TH-1 cytokines, Interferon gamma, interleukin-6, interleukin-12p40, tumor necrosis factor, soluble CD40 ligand, and Interleukin-1*β* and anti-inflammatory TH-2 cytokines, Interleukin-4, Interleukin-RA, Interleukin-10, and Interleukin-13 in overweight and obese chimpanzees. We also observed increased levels of metabolic hormones glucagon-like-peptide-1, glucagon, connecting peptide, insulin, pancreatic peptide YY_3–36_, and leptin in the plasma of overweight and obese chimpanzees. Chemokine, eotaxin, fractalkine, and monocyte chemoattractant protein-1 were higher in lean compared to obese chimpanzees, while chemokine ligand 8 increased in plasma of obese chimpanzees. We also observed an obesity-related effect on immune function as demonstrated by lower mitogen induced proliferation, and natural killer activity and higher production of IFN-*γ* by PBMC in Elispot assay, These findings suggest that lean, overweight, and obese chimpanzees share circulating inflammatory cytokines and metabolic hormone levels with humans and that chimpanzees can serve as a useful animal model for human studies.

## 1. Introduction

Obesity in humans has been established as a risk factor for a multitude of maladies including cardiovascular disease (CVD), Type 2 diabetes mellitus (T2DM), hypertension, renal disease, and neurologic dysfunction. Furthermore, obesity has been causally linked to a variety of cancers either as a risk factor or as a negative factor for prognosis [1–3].

Recent studies have suggested that the link to these disorders or diseases is due to a chronic low-grade inflammation that is associated with obesity [4–7]. Debate exists whether the inflammation is a product of obesity or rather inflammation results in an obese state. Nevertheless, once excess adipose tissue is established, the functions of the tissue appear to be comparable with a dynamic endocrine organ [8, 9]. In the obese individual, macrophages, adipocytes, and epithelial cells communicate via obesity-associated hormones, inflammatory cytokines, and other mediators. For example, adipose tissue is known to produce and secrete various adipokines, such as leptin and adiponectin, and pro-inflammatory factors, TNF, IL-6, IL-1, and C-reactive protein (CRP) [4, 5]. All of these factors not only are important in adipogenesis but have been strongly linked to the onset of CVD, T2DM [1–3], and metabolic syndrome [6, 7]. In addition, recent reports have associated this chronic inflammation seen in obesity with cancer promotion and development [10–12]. A longitudinal community-based Cardiovascular Risk Factors Prevalence Study (CRISPS) in human subjects from Hong Kong shows that IL-6, soluble tumor necrosis factor receptor 2 (sTNFR2; as a surrogate marker of tumor necrosis factor-α activity), leptin, lipocalin 2, adiponectin, and adipocyte-fatty acid binding protein (A-FABP) are predictors for cancer development [10–12].

In the last decade, several studies have shown elevated IL-6, TNF, and IL-1 levels in obese patients [13–17]; however, data regarding the relevance of these cytokines are controversial [18, 19]. This could be partially attributable to the complex etiology of obesity, which consists of an interaction between genetics, diet, and physical activity levels, and is additionally influenced by environmental, socioeconomic, and behavioral factors.

Chimpanzees have the closest homology to humans and also share a multitude of similar diseases related to obesity including CVD, T2DM, hypertension, and renal disease. Obesity in captive chimpanzees is a known management problem. Although understood in humans, it is unknown if obese chimpanzees share a chronic inflammatory state. The study of obese chimpanzees could both help in the management of this species and also lend clues to the human obesity epidemic.

In the present study, we examined cytokine, chemokine, and metabolic hormones levels in plasma of three chimpanzee weight categories; lean, overweight, and obese. We measured plasma concentrations of the following cytokines and chemokines: Interferon gamma (IFN-*γ*), interleukin-6 (IL-6), interleukin-12p40 (IL-12p40), tumor necrosis factor (TNF), soluble CD40L (sCD40L), interleukin-1*β* (IL-1*β*), interleukin-4 (IL-4), interleukin-RA (IL-RA), interleukin-10 (IL-10), interleukin-13 (IL-13), eotaxin, fractalkine, monocyte chemoattractant protein-1(MCP-1) and chemokine (CXC motif) ligand8 (CXCL8 or formally known as IL-8)), and metabolic hormones such as connecting peptide (c-peptide), Glucagon-like-peptide-1 (GLP-1), glucagon, insulin, peptide tyrosine or pancreatic peptide YY_3–36_ (PYY), and leptin. We also measured the influence of obesity on immune function. The purpose of this study is to investigate the relationship between obesity and these cytokine/chemokine/metabolic hormones in chimpanzees. The hypothesis is that chimpanzees will be similar to humans and will demonstrate an inflammatory profile with increasing adiposity.

## 2. Methods

### 2.1. Study Groups

The study population was grouped according to the body condition scoring and defined as lean, overweight, and obese. Chimpanzees in lean group were defined as muscular body, normal body condition, some abdominal tuck, and neither concave nor convex abdomen. Overweight chimpanzees were defined as round convex abdomen, big thighs, and the presence of fat around gluteal muscles. Obese chimpanzees were defined as having a very large abdomen that extends outside of body frame, pectoral fat, the presence of fat around gluteal muscles, and fatty deposits in axillary regions and/or below biceps. Blood samples were collected at same time points during the 12-month period. All subjects were considered healthy and in their normal social groups at the time they were sampled. Chimpanzees with chronic diseases were not included in this study. The study population consisting of chimpanzees used in the study is shown in Table 1.

All chimpanzees are group housed (multimale, multifemale) at the Michale E. Keeling Center for Comparative Medicine and Research at the University of Texas MD Anderson Cancer Center and maintained in accordance with the “Guide for the Care and Use of Laboratory Animals” of the Institute of Laboratory Animal Resources, National Research Council. The facility is fully accredited by the Association for the Assessment and Accreditation of Laboratory Animal Care International. All chimpanzee enclosures are cleaned daily and all chimpanzees receive a nutritionally complete diet. Chimpanzees have full access to water and have indoor and outdoor access. The primary source of nutrition is Harlan Teklad Chimpanzee diet (#7775), a high fiber, 20% protein-containing diet that is fed twice a day. In addition, chimpanzees are fed four produce meals each day. Environmental enrichment is provided daily using various food puzzle devices, forage such as popcorn or sunflower seeds scattered in the grass of outdoor enclosures, manipulable items such cardboard boxes and through treats given as rewards for positive reinforcement training.

### 2.2. Blood Collection and PBMCs Preparation

Blood sample (10 mL) was collected in heparin coated collection tubes and immediately plasma was separated by centrifugation and stored at −80°C until further use. Peripheral blood mononuclear cells (PBMCs) were separated from heparinized blood by centrifugation through a Histopaque (density, 1.077 g/mL; Sigma, St Louis, MO). Peripheral blood mononuclear cells were removed from the interface and washed twice with complete RPMI 1640 (Hyclone, Logan, UT) supplemented with 100 000 U/L penicillin (Sigma), 100 mg/L streptomycin (Sigma), 2 mmol/L L-glutamine (Sigma), and 25 mmol/L HEPES (Sigma). Cells were resuspended in appropriate concentrations in complete RPMI for cultures in various immune assays.

### 2.3. Circulating Plasma Cytokine, Chemokine, and Metabolic Hormones Quantification

Plasma concentration of cytokines/chemokines, IFN-*γ*, IL-6, IL-12p40, TNF, sCD40L, IL-1 *β*, IL-4, IL-10, IL-13, IL-RA, MCP-1, fractakine, eotaxin, IL-15, IL-17A, TGF-α, macrophage inflammatory proteins-1α, (MIP-1α or CCL3), Macrophage inflammatory proteins-1*β* (MIP-1*β* or CCL4), and RANTES and metabolic hormone panel containing leptin, C-peptide, GLP-1 (active) glucagon, insulin, pancreatic peptide, and PYY (total) were measured using the Multiplex MAP Magnetic Bead-based immunoassay kits (Millipore Corp. Billerica, MA) or kits from Invitrogen (Carlsbad, CA). Blood was collected from lean (*n* #28), overweight (*n* #10), and obese (*n* #10) chimpanzees. Following centrifugation, plasma was aliquoted immediately and frozen at −80°C until assayed. On the day of assay, frozen plasma was thawed, mixed by vortexing, and then centrifuged at 10,000 rpm for 5 min to isolate debris prior to use in the assay. All assays were conducted according to the manufacturer's instructions using handheld magnetic separator block for 96-well flat bottom plates (Millipore, Millipore Corp.) and analyzed using the Luminex 200 system (Bio-Rad Corp.). All samples were run in duplicate and cytokine standards supplied by the manufacturer were run on each plate. Acquisition gates were set at 8,000–15,000, sample volume was 25 *μ*L, and 50 events per bead were acquired. Mean fluorescence intensity was analyzed using the BioPlex manager software version 5.0 (Bio-Rad) and compared to a standard curve to generate concentration values. Values below the range of the standard curve were set to the lower limit of detection. The assay sensitivities (minimum detectable concentrations, pg/mL) were for IFN-*γ* and TNF (0.1 pg/mL), IL-6 and IL-10 (0.3 pg/mL), for IL-1*β* and IL-13 (0.4 pg/mL), for IL-12p40 (10.5 pg/mL), for sCD40L (4.9 pg/mL), for IL-4 (0.6 pg/mL), for IL-RA (2.9 pg/mL), for Eotaxin (1.2 pg/mL), for Fractakine (6.0 pg/mL), for MCP-1 (0.9 pg/mL), for CXCL8 (0.2 pg/mL), for C-peptide (24 pg/mL), for GLP-1 (7.0 pg/mL), for Glucagon (6.0 pg/mL), for Insulin (58 pg/mL), for leptin (27.0 pg/mL), and for PYY (8.0 mg/mL).

### 2.4. Lymphocyte Proliferation

The proliferation of PBMCs samples from the chimpanzees was determined by the standard [^3^H] thymidine incorporation as described previously [20, 21]. Briefly, aliquots of the PBMCs (10^5^/well) were seeded in triplicate wells of 96-well plates and stimulated for 6 days individually with the mitogens concanavalin-A (Con A), phytohemagglutinin (PHA), and pokeweed mitogen (PWM) (each at 5 *μ*g/mL final concentration) (Sigma, St Louis. MO). The culture medium without added mitogens served as negative control. After culturing for 5 day at 37°C in 5% CO_2_, each well was pulsed for 18 h with 0.1 *μ*Ci of methyl-^3^H-thymidine (ICN, Irvine, CA). These mitogen concentrations, PBMCs numbers, and incubation times were found to be optimal conditions for stimulation of PBMCs from healthy animals in our laboratory. The contents of the wells were then harvested onto glass fiber discs using a Skatron cell harvester (Skatron Laboratories, VA, and USA). The amount of radioactivity was determined in a Wallac Liquid Scintillation Counter (Wallac1409, Mustionkatu, Tarku, Finland). The results were reported as corrected counts per minute (cpm), which is the average count per minute (cpm) of mitogen-stimulated cultures minus the average cpm of cultures without mitogen.

### 2.5. ELISPOT Assay for Detecting Antigen-Specific IFN-*γ* Producing Cells

Freshly-isolated PBMCs, as described above, were stimulated with the mitogens PHA, Con A, and PWM (each at 5 *μ*g/mL final concentration) to determine the numbers of IFN-*γ*-producing cells by the Enzyme Linked Immuno Spot (ELISPOT) assay using the methodology reported earlier [20, 22]. Briefly, aliquots of PBMCs (10^5^/well) were seeded in triplicate wells of 96-well plates (polyvinylidene difluoride backed plates, MAIP S 45, Millipore, Bedford, MA) precoated with the primary IFN-*γ* antibody and the lymphocytes were stimulated with the different mitogens. After incubation for 30 hr at 37°C, the cells were removed and the wells were thoroughly washed with PBS and developed as per protocol provided by the manufacturer. Purple colored spots representing individual cells secreting IFN-*γ* were counted by an independent agency (Zellnet Consulting, New Jersey, NJ) using the KS-ELISPOT automatic system (Carl Zeiss Inc., Thornwood, NY) for the quantitative analysis of the number of IFN-*γ* spot forming cells (SFC) for 10^5^ input PBMCs. Responses were considered positive when the numbers of SFC with the test antigen were at least five and also were five above the background control values from cells cultured in the medium alone.

### 2.6. *Ex Vivo *Induction of Cytokines by TLR Ligands

PBMCs obtained after centrifugation of blood using a density gradient were washed with PBS. Aliquots of 1 × 10^5^ cells resuspended in culture medium RPMI-1640 (Hyclone Laboratories) were dispensed in each well of a 96-well plate. The culture medium used was free of detectable endotoxin (<0.1 EU/mL) and all other solutions were prepared using pyrogen-free water and sterile polypropylene plastic ware. The cells were then incubated with or without TLR ligand peptidoglycan (PGN, TLR-2 ligand), ultrapurified lipopolysaccharide (LPS, TLR-4 ligand), polyinosinic-polycytidylic acid (poly IC, TLR-3 ligand), and cytosine-phosphate-guanine (CpG) DNA (TLR-9 ligand) all from Invivogen (Invivogen Corp., San Diego, CA, USA) at 1 *μ*g/mL each for 24 hr at 37°C in a 5% CO_2_ atmosphere. The cell-free supernatant was harvested and stored at −70°C for subsequent assays of cytokines and chemokines with the human inflammation cytometric bead array (CBA) kit as described above.

### 2.7. Natural Killer Cytotoxic Assay

Natural killer cells activity was assessed using the standard 4 h radioactive chromium (^51^Cr-)release assay as described previously [20]. Peripheral blood mononuclear cells were isolated from heparinized blood and incubated with Chromium 51 (^51^Cr-) labeled K562 target cells to yield 100 : 1, 50 : 1, 25 : 1, and 12.5 : 1 effector-to-target ratios. The assay was performed in triplicate in U-bottom microtiter plates. The microtiter plates were then incubated for 4 hr at 37°C in a 5% carbon dioxide incubator. At the end of the incubation, 100 *μ*L of supernatant was collected from each well and the amount of ^51^Cr released was determined using the *γ*-counter. To account for the maximum release, the cells were incubated with 5% Triton X-100. Spontaneous release was determined from target cells incubated without added effector cells. The % of specific lysis was calculated using the mean counts per minute (cpm) by the following formula: % Specific lysis = (experimental release − spontaneous release)/(maximum release − spontaneous release) × 100.

### 2.8. Statistical Analysis

For statistical analysis, samples were grouped according to body condition of the animals from which samples were obtained. Comparison between groups of chimpanzees was done by one-way analysis of variance with the Kruskal-Wallis test and Gaussian approximation with Dunn's multiple comparison tests. Data are presented as group means ± SD. Only differences with a probability less than 0.05 were considered to be significant. All statistical analyses were conducted using GraphPad Prism 6.00 (GraphPad Software, San Diego, California, USA).

## 3. Results

In the present study, we compared the circulating plasma concentration of proinflammatory cytokines, anti-inflammatory cytokines, growth factors, and metabolic hormones in lean, overweight, and obese chimpanzees.

### 3.1. Inflammatory Cytokines and Metabolic Hormones

**Figure 1:**
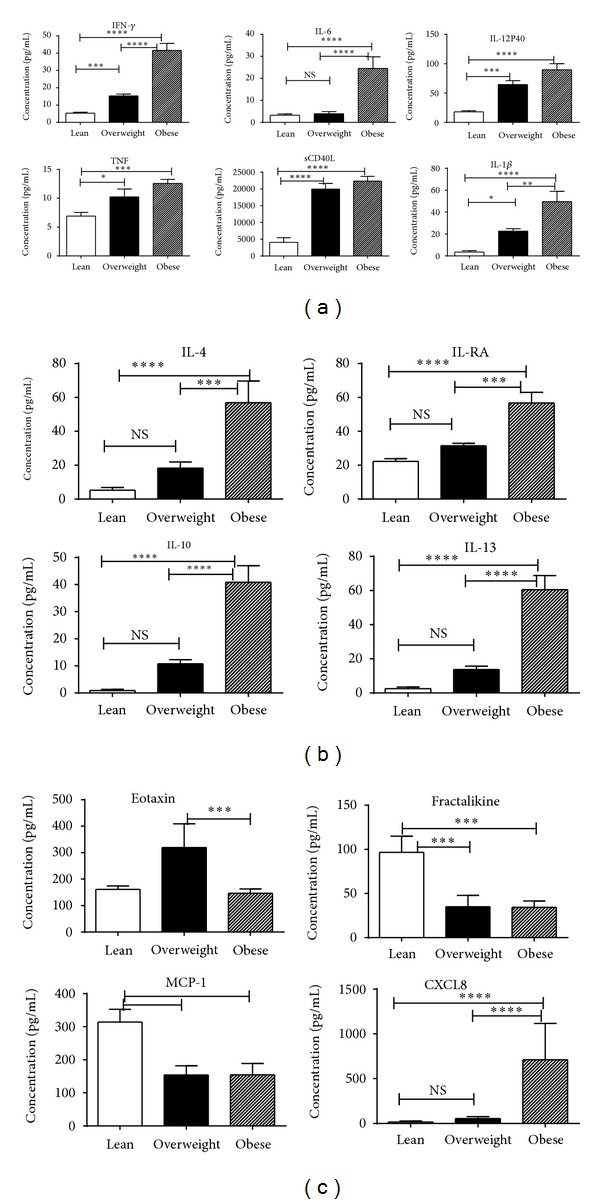
Measurement of cytokines by multiplex cytokine bead array (CBA) in plasma of lean (*N* #28), overweight (*N* #10), and obese (*N* #10) chimpanzees using Bio-Rad 200, Luminex technology. The results are expressed as pg/mL concentration and are an average of two distinct experiments. (a) Shows proinflammatory cytokines, (b) anti-inflammatory cytokines, and (c) chemokine. See method section for experiment details. Comparison between groups of chimpanzees was done by one-way analysis of variance with the Kruskal-Wallis test and Gaussian approximation with Dunn's multiple comparison tests. Data are presented as group means ± SD. Only differences with a probability less than 0.05 were considered to be significant. All statistical analyses were conducted using GraphPad Prism 6.00 (GraphPad Software, San Diego, California, USA).

Plasma IFN-*γ*, IL-6, IL-12p40, TNF, sCD40L, and IL-1*β* levels were found to be significantly higher (*P* < 0.0005) in overweight and obese chimpanzees than in lean controls (Figure 1(a)). Anti-inflammatory cytokines IL-4, IL-10, IL-13, and IL-RA were also significantly higher in overweight and obese chimpanzees (Figure 1(b)).

Plasma MCP-1 and fractakine levels were significantly higher in the lean group relative to the obese group while eotaxin levels were higher in the overweight group compared to the obese group; however, no difference was found between obese and lean group (Figure 1(c)). Cytokine CXCL8 was significantly higher in the obese group compared to lean and overweight group (Figure 1(c)).

**Figure 2:**
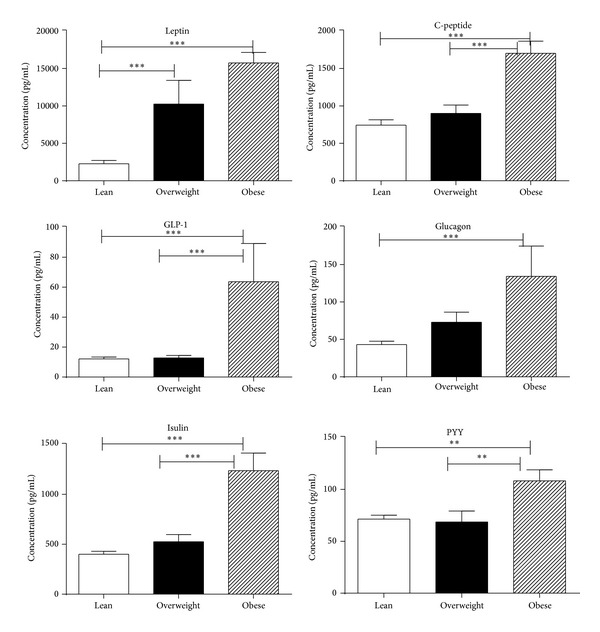
Metabolic hormones were determined multiplex cytokine bead array (CBA) in plasma of lean, overweight, and obese chimpanzee using Bio-Rad 200, Luminex technology. The results are expressed as pg/mL concentration. The results shown are an average of two distinct experiments. Comparison between groups of chimpanzees was done by one-way analysis of variance with the Kruskal-Wallis test as described in Figure 1.

Moreover, no significant differences were observed in IL-12, IL-15, IL-17A, TGF-α, MIP-1a, MIP-1*β*, and RANTES plasma concentrations between the 3 groups of chimpanzees (data not shown). Obesity was also associated with increased levels of leptin, insulin, glucagon, GLP-1, PYY, and C-peptide in overweight and obese chimpanzees (Figure 2).

### 3.2. Mitogen Induced Proliferation and IFN-*γ* Production

**Figure 3:**
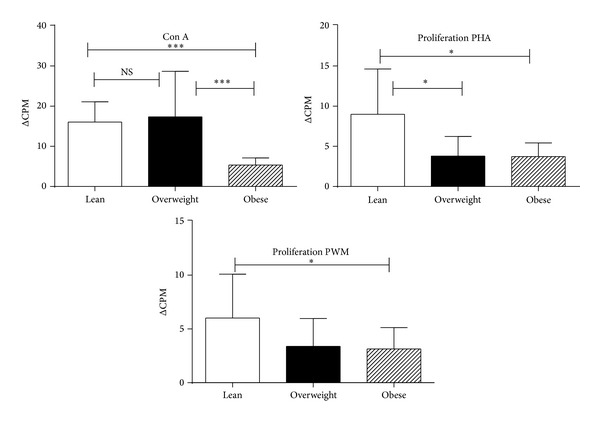
Proliferative response of PBMCs to mitogens in lean, overweight, and obese chimpanzees. PBMCs isolated from the blood samples of the chimpanzees were used for determining proliferative response in triplicate wells using the standard [^3^H] thymidine incorporation assay. The proliferation responses are expressed as delta (Δ) count per minuets (CPM). Δ value representing increase in radioactivity incorporated in presence of the mitogen over to that in medium control. The results shown are average of three distinct experiments and comparison between groups of chimpanzees was done by one-way analysis of variance with the Kruskal-Wallis test as described in Figure 1.

We also studied the immune responses of lean, overweight, and obese chimpanzees to test the hypothesis that obesity is associated with an impaired immune function and deregulated inflammatory response. In vitro proliferative responses of PBMCs and the production of IFN-*γ* were determined. We observed ConA, PHA, and PWM, 3 different mitogens inducing proliferative response of lymphocytes significantly lower in the overweight and obese groups compared to the lean group (Figure 3).

**Figure 4:**
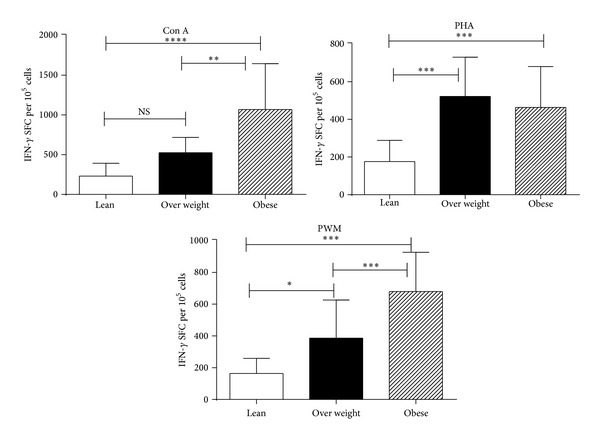
IFN-*γ* Elispot response to mitogens in lean, overweight, and obese chimpanzees. PBMCs isolated from the blood samples of the chimpanzees. Triplicate wells of the 96-well microtiter plates, precoated with IFN-*γ* antibody, were seeded with 10^5^ PBMCs from the monkeys and incubated with 5 *μ*g of each of the mitogens for 36 h at 37°C. At the end of the incubation period, the wells were washed and stained with biotinylated second IFN-*γ* antibody. The total number of spot forming cells in each of the mitogen-stimulated wells was counted and adjusted to control medium as background. See method section for experiment details. The results shown are an average of two distinct experiments and comparison between groups of chimpanzees was done by one-way analysis of variance with the Kruskal-Wallis test as described in Figure 1.

In IFN-*γ* producing T cells in IFN-*γ* Elispot assay, PHA, Con A, and PWM stimulated PBMCs from the obese and overweight groups produced significantly higher levels of IFN-*γ*  (*P* < 0.005) than those from the lean group (Figure 4).

### 3.3. Natural Killer Cytotoxic Assay

**Figure 5:**
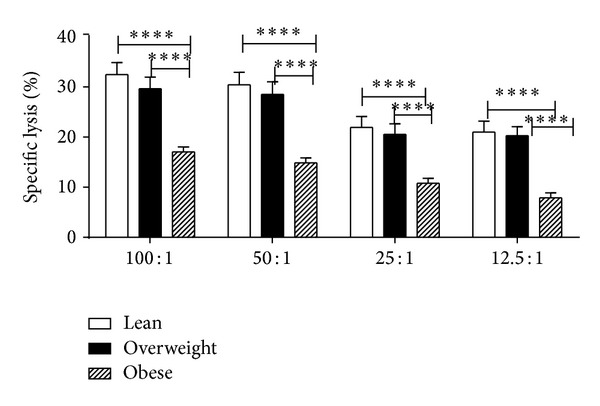
Measurement of natural killer cell activity in lean, overweight, and obese chimpanzees. PBMCs isolated from the blood samples of the chimpanzees were used for determining NK activity in triplicate wells using the standard ^51^Cr release assay. Results are expressed as % specific lysis. The values shown were obtained after subtracting the background lysis of control target cells (K652) from that of target cells. The data presented are averages from three different experiments.

We also studied natural killer (NK) cells activity in lean, overweight, and obese chimpanzees using standard ^51^Cr release assay. We observed significantly lower NK activity (47% compared to 51%) in obese chimpanzees compared to lean chimpanzees (Figure 5).

### 3.4. PBMCs Stimulated with TLR Ligands

**Figure 6:**
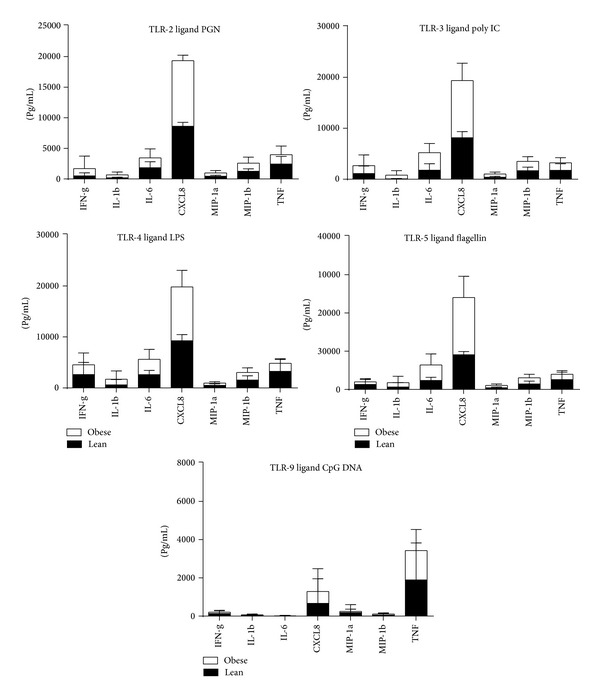
Measurement of cytokines in PBMCs stimulated with TLR ligands. PBMCs isolated from lean and obese chimpanzee blood were stimulated with TLR ligands for 20 hr and supernatants were measured for cytokines using Bio-Rad 200, Luminex technology as described above. The results are expressed as pg/mL concentration. The results shown are an average of two distinct experiments. Comparison between groups of chimpanzees was done by one-way analysis of variance with the Kruskal-Wallis test as described above.

TLRs play a crucial role in host defense against invading pathogens by mediating innate and adaptive immunities. Recent studies suggest that adipocytes may play an important role in the physiological regulation of immune responses in fat deposits via toll-like receptor (TLR) signaling cascades [23]. Therefor we decided to study effect of various TLR ligands on PBMCs isolated from different groups of chimpanzees. PBMCs isolated from lean and obese chimpanzee blood were stimulated with TLR ligands for 20 hr and supernatants were measured for cytokines. Obese chimpanzees showed increased capability to produce IL-6 and CXCL8 after TLR-2, TLR-3, TLR-5, and TLR-9 stimulation (Figure 6). In contrast, when PBMCs were stimulated with TLR-4, the obese chimpanzees had decreased IL-6 and CXCL8 production compared to lean chimpanzees. However, no statistical difference was found between lean and obese chimpanzees for production of inflammatory cytokines by PBMCs stimulated with lipopolysaccharide (TLRs).

## 4. Discussion

Epidemiologic evidence suggests an important link between obesity and inflammation, although these findings were not appreciated in terms of the pathophysiologic conditions associated with obesity. For example, the levels of circulating fibrinogen and other acute phase reactants, including TNF, IL-6, and C-reactive protein (CRP) were found to be elevated in obesity [24, 25]. In the present study we investigated the relationship between cytokines and obesity by measuring plasma concentrations of different cytokines and metabolic hormones in lean, overweight, and obese chimpanzees. Furthermore, we also examined the influence of obesity on immune function.

The important finding from the present study is that obesity is associated with simultaneous induction of several cytokines and metabolic hormones in plasma of overweight and obese chimpanzees compared to lean counterparts. We demonstrated that obesity is associated with increased levels of C-peptide, leptin, insulin, GLP-1, PYY, and glucagon. Our findings are in agreement with the studies demonstrating positive relationships between levels of adipose tissue and circulating plasma C-peptide, insulin, leptin, IL-6, and C-reactive protein levels and release of adipokines by adipose tissue matrix of obese humans [26–28].

The plasma C-peptide has proved to be extremely valuable in the study of the natural history of type 1 diabetes to monitor insulin secretion in patients with insulin antibodies and as an adjunct in the investigation of patients with hypoglycemic disorders [29]. Abu-Farha et al. [24] have shown in a human study that serum leptin concentrations rise in proportion to the body adiposity and therefore, obese individuals with metabolic syndrome generally have higher circulating leptin concentrations. In animal models, increased expressions of leptin are associated with release of proinflammatory cytokines such as TNF and IL-6 in monocytes and macrophages. Leptin also has a significant effect on adaptive immunity, such as inducing a switch toward Th1-cell mediated immune responses by increasing IFN-*γ*, TNF secretion, and the suppression of Th2-cell responses in adipose tissue [30, 31].

Glucagon-like peptide 1 (GLP-1), an insulinotropic hormone, also increased with obesity in our study. GLP-1 has been shown to inhibit food intake and induce weight loss in humans independent of type 2 diabetes status. The anorectic effect of GLP-1 could be attributable to both its effect on gastric emptying and a direct effect on neurons in the central nervous system involved in appetite regulation [32]. GLP-1 colocalizes with PYY in the endocrine L cells in the ileal mucosa [33]. Similar to GLP-1, PYY levels increase postprandially and act as an appetite suppressant [34].

Overweight and obese chimps also had elevated levels of IFN-*γ*, TNF, IL-6, CXCL8, IL-12p40, sCD40L, IL-1*β*, IL-4, IL-RA, IL-10, IL-13, and MCP-1 compared to the lean counterparts. These findings are consistent with the recent reports in a human study by Jung et al. [35], demonstrating the effect of weight loss on serum cytokines in human obesity. Other studies have also revealed that proinflammatory cytokines IL-6 and TNF were among the first to be implicated as a predictor or pathogenic mediator of insulin resistance, CVD, and in patients with type 2 diabetes [36, 37]. Bruun et al. [38] found that plasma levels of CXCL8 were higher in obese subjects compared with lean subjects. This finding could be related to the fact that obesity is known to be associated with a chronic state of low-grade inflammation [39].

Our study showed IL-*β*, IL-6, and IL-10 were higher in obese chimpanzees. IL-1*β* together with IL-6 concentrations reportedly predicts risk for type 2 diabetes in humans better than either cytokine alone [40]. IL-10 is an anti-inflammatory cytokine produced by immune cells in adipose tissue that acts on adipocytes to improve insulin signaling, potentially decreasing further macrophage recruitment [41, 42]. In 2003, Esposito et al. [43] showed that circulating levels of IL-10 were elevated in obese women and speculated that the higher IL-10 levels represented the body's attempt to inhibit continued proinflammatory cytokine production. In our study, we also found significantly increased levels of IL-10 in overweight and obese chimpanzees. Esposito et al. [43] also reported that changes in life style aimed at reducing body weight and increasing physical activity over 1 year significantly reduced high IL-10 levels in obese women, while Manigrasso et al. [44] reported no significant change in IL-10 levels after an observed reduction in body weight in another obesity study.

In the present study IL-12, MCP-1, and sCD40L levels were elevated in obese chimpanzees. Strissel et al. [45] owed experimental evidence from high-fat-diet-fed mice suggesting that IL-12 could have an additional role in the systemic low-grade inflammation and the ensuing development of obesity-related insulin resistance. Schernthaner et al. [46] demonstrated circulating sCD40L levels in obese patients were closely related to insulin levels, triglycerides, and to the inflammatory biomarkers (MCP-1). Two independent studies convincingly showed that the adipocyte-specific overexpression of MCP-1 in mice was sufficient to increase macrophage recruitment to adipose tissue and cause systemic insulin resistance, hepatic steatosis, and insulin resistance in liver and muscle [47].

Data from this study indicate obesity is related to lower T- and B-cell proliferation function and activity of natural killer cells and higher IFN-*γ* secretion in obese compared to lean chimpanzees. Obesity has been linked to impaired T- and B-cell function, suggesting that metabolic alterations can be induced by or are a consequence of an altered self-immune tolerance and/or modulation of immune responses as reported in obese men and women who were compared with nonobese control subjects [48]. Obese children and adolescents have been reported to have impaired cutaneous delayed-type hypersensitivity responses, mitogen-stimulated lymphocyte proliferation, and bactericidal capacity [49, 50]. Few reports exist, comparing activity of natural killer cells in obese and nonobese human beings. Moriguchi et al. [51] showed that obesity is related to reduced activity of natural killer cells in older men and women. Similar results were reported in obese Zucker rats; activity of natural killer cells has been reported to be suppressed, an effect found to be reversible through exercise training via improved lymphocyte glucose uptake and enhanced GLUT-1 expression [52].

We have observed decreased cytokines (IL-6 and CXCL8) in obese chimpanzees when PBMCs were stimulated with specific ligands for TLR3, TLR4, and TLR9 but differences were not significant compared to lean chimpanzees. Recently, Kopp et al. [53] demonstrated that IL-6 release is stimulated in murine adipocytes by specific ligands for TLR1/2, TLR2/TLR6, TLR3, and TLR4, whereas monocyte chemoattractant protein-1 (MCP-1) release is increased upon stimulation with specific ligands for TLR1/2, TLR3, and TLR4.

An advantage of the chimpanzee model for obesity and inflammatory studies, besides a genomic similarity greater than 98% in comparison to humans, is that we can control obesity-related exogenous factors, such as nutrition and lifestyle, thus studying the molecular events leading to metabolic syndrome, inflammatory, and/or autoimmune conditions. We are aware that our study has limitations. One of these limitations is that we are reporting systemic circulating cytokines/chemokines and metabolic hormones concentrations. For example, leptin function may be modulated by local leptin concentration, the ratio between free and bound leptin, the expression of different forms of the receptors, the ratio between signaling and nonsignaling receptors, and/or the presence of specific inhibitors. A second limitation is that we have a limited number of overweight and obese animals in our chimpanzee colony from which to acquire samples.

The most important finding of the present study is that plasma cytokines/chemokines and metabolic hormones concentrations are significantly increased in obese chimpanzees relative to normal-weight healthy chimpanzees. Also, in the present study, we observed an influence of obesity on immune function. In fact, the current study provides evidence that a proinflammatory state could be regarded as a significant prognostic indicator of the risk of obesity, CVD, and metabolic syndrome. Although obesity is a complex, multifactorial trait that cannot be explained by one factor, the findings of the present study represent important directions for the future planning of programs designed to prevent obesity-related diseases and in the identification of novel antiobesity targets.

## Figures and Tables

**Table 1:** Distribution of age and sex of the chimpanzees in the study.

Study groups	Female		Male	
	*N*	Age (years)∗	*N*	Age (years)∗
Lean	15	29.8 ± 10.5	13	23.4 ± 6.9
Overweight	6	29.5 ± 6.25	4	18.25 ± 0.96
Obese	8	25.11 ± 3.51	2	22.5 ± 0.71

*Mean ± S.D.
